# Sex‐related changes in lactate dehydrogenase A expression differently impact the immune response in melanoma

**DOI:** 10.1111/febs.17423

**Published:** 2025-01-31

**Authors:** Marta Iozzo, Giuseppina Comito, Luigi Ippolito, Giada Sandrini, Elisa Pardella, Erica Pranzini, Mariaelena Capone, Gabriele Madonna, Paolo Antonio Ascierto, Paola Chiarugi, Elisa Giannoni

**Affiliations:** ^1^ Department of Experimental and Clinical Biomedical Sciences University of Florence Italy; ^2^ Institute of Oncology Research (IOR) Università della Svizzera Italiana (USI) Bellinzona Switzerland; ^3^ Istituto Nazionale Tumori‐IRCCS‐Fondazione G. Pascale Naples Italy

**Keywords:** CD8^+^, lactate, melanoma, sex, Treg

## Abstract

Melanoma is more aggressive in male patients than female ones and this is associated with sexual dimorphism in immune responses. Taking into consideration the impact tumour metabolic alterations in affecting the immune landscape, we aimed to investigate the effect of the sex‐dependent metabolic profile of melanoma in re‐shaping immune composition. Melanoma is characterised by Warburg metabolism, and secreted lactate has emerged as a key driver in the establishment of an immunosuppressive environment. Here, we identified lactate dehydrogenase A (LDH‐A) as a crucial player in modulating sex‐related differences in melanoma immune responses, both *in vitro* and in patient‐derived specimens. LDH‐A is associated with higher lactate secretion in male melanoma cells, which leads to a significant enrichment in pro‐tumoural regulatory T cells (Treg) with a concurrent decrease in the number and activity of anti‐tumour CD8^+^ T cells. Remarkably, pharmacological and genetic impairment of LDH‐A in male melanoma cells normalises Treg and CD8^+^ infiltration. In keeping with this, *in vivo* pharmacological targeting of LDH‐A in melanoma‐bearing male mice impairs tumour growth and lung colonisation, with a concomitant modulation of Treg and CD8^+^ T cells infiltration. Taken together, our findings highlight the sex‐related differences promoted by LDH‐A in immune reshaping in melanoma, and suggest that therapeutic targeting of LDH‐A could be leveraged as an effective strategy to abolish the sex‐gap in melanoma progression.

## Graphical abstract

Here, we explore a differential sex‐related metabolic‐immune relationship in melanoma. Melanoma tumours in male patients exhibit a higher LDH‐A‐driven production of lactate compared to their female counterparts. In turn, lactate acts as an immunosuppressive molecule, promoting pro‐tumoural Treg cells and suppressing anti‐tumoural CD8^+^ T‐cell activity. Targeting LDH‐A in male preclinical models restores immune composition, dampens tumour growth and metastatic dissemination. Thus, LDH‐A emerges as a promising target in a sex‐dependent context in melanoma.

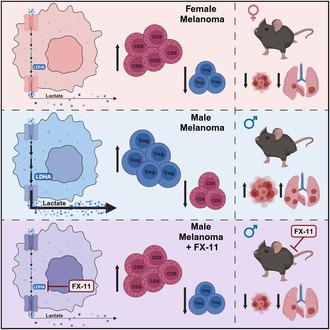

**Abbreviations**
CTLs: cytotoxic T lymphocytes
DCs: dendritic cells
ERRα: oestrogen‐related receptor alpha
FoxP3: forkhead box P3
GSEA: gene set enrichment analysis
GZMB: granzyme B
HKII: hexokinase II
LDH‐A: lactate dehydrogenase A
MCTs: monocarboxylate transporters
MDSCs: myeloid‐derived suppressor cells
NK cells: natural‐killer cells
PBMCs: peripheral blood mononuclear cells
PRF1: perforin
TMA: tissue microarray
TME: tumour microenvironment
TNF‐α: tumour necrosis factor α
Treg: regulatory T cells

## Introduction

Melanoma is the most lethal form of skin cancer worldwide. Epidemiological studies have shown that sex disparities are particularly accentuated in melanoma, where male sex is associated with increased incidence and mortality [1]. Indeed, after a diagnosis of melanoma, women generally have a more favourable outcome than men, with longer disease‐free time before relapses and lower mortality rate [1, 2]. In this scenario, gender disparity in immune responses has recently emerged as a driving determinant in melanoma incidence, outcome and therapy response [3, 4], with females generally showing stronger immune responses [5, 6].

The metabolic profile of tumour cells and cancer‐associated cell populations within the tumour microenvironment (TME) strongly affects the functionality of the immune components [7, 8, 9]. Indeed, the metabolic signature of infiltrating immune cells, i.e., CD4^+^, CD8^+^ cells, M1/M2 macrophages, natural‐killer cells (NK cells), dendritic cells (DCs) and myeloid‐derived suppressor cells (MDSCs), is shaped not only by cytokines and chemokines but also by several metabolites released by tumour and tumour‐associated cells, such as kynurenine, arginine and adenosine [10, 11].

In this regard, melanoma cells mostly rely on a glycolytic metabolism displaying high glucose intake and fermentation of glucose to lactate. This metabolic profile is supported by the overexpression of several glycolytic enzymes, such as hexokinase II (HKII) and lactate dehydrogenase A (LDH‐A), that mediates the conversion of pyruvate into lactate, which is continuously exported in the extracellular environment by monocarboxylate transporters (MCTs). This leads to TME acidification playing a crucial role in immune escape in melanoma [12, 13, 14]. Indeed, lactate can contribute to tumour evasion by altering the metabolism and function of cytotoxic T lymphocytes (CTLs) [15], concurring to macrophage polarisation towards a pro‐tumoural M2‐like phenotype [16, 17, 18] and favouring an enrichment in CD4 immunosuppressive Tregs [11, 19, 20].

Although several studies previously demonstrated gender differences in melanoma progression [4], metabolic mechanisms influencing sex‐dependent regulation of the immune component have not yet been defined.

Herein, we investigated how metabolic sex disparities could impact on the reshaping of the tumour immune‐infiltrate composition and influence the establishment of an immunosuppressive environment in melanoma.

## Results

### Male‐derived melanoma exhibits a pronounced glycolytic profile and high lactate secretion

It has been extensively reported that melanoma cells exhibit a Warburg metabolism [13, 21], characterised by a high extracellular accumulation of lactate as the end‐product of aerobic glycolysis [22].

**Fig. 1:**
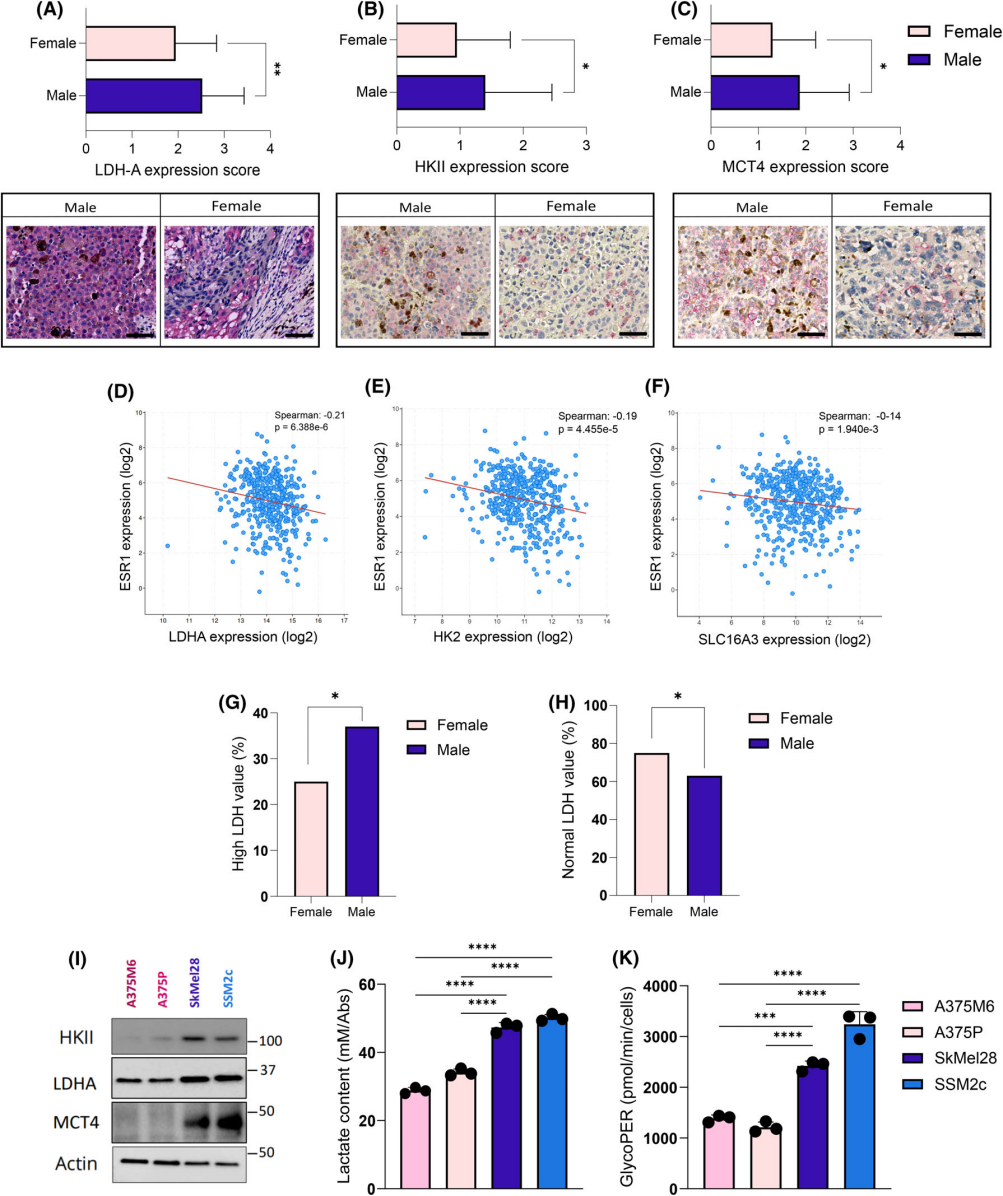
Male‐derived melanoma exhibits a pronounced glycolytic metabolism, establishing a lactate‐rich environment. (A–C) Expression score of LDH‐A (A), HKII (B) and MCT4 (C) in representative tissue cores from TMA (magnification 40×; scale bar: 20 μm) including male and female melanoma samples; *n* = 80. (D–F) Inverse co‐expression between *LDH‐A*, *HK2* and *SLC16A3* with *ESR1* as indicated in cBioPortal TCGA melanoma databases; *n* = 448. (G, H) Analysis of LDH serum level in male and female melanoma patients; *n* = 114. (I) Representative western blot images of the indicated glycolytic markers, performed on female (A375P, A375M6) and male (SkMel28, SSM2c) melanoma cell lysates. β‐Actin was used as a loading control. (J) Extracellular lactate levels measured by enzymatic assay with Lactate Assay Kit following 48 h of cell culture; *n =* 3. (K) Basal glycolysis detected with Seahorse glycolytic rate assay. Each dot represents an independent experiment. Data information: Error bars represent means ± SEM of *n* independent experiments. Statistical analysis was performed using two‐tailed unpaired Student's *t*‐test (A–C, G, H) and one‐way ANOVA followed by Tukey's test (J, K). *P < 0.05; **P < 0.005; ***P < 0.001; ****P < 0.0001.

To investigate the existence of a sex‐related difference in the reliance of melanoma cells on glycolysis, we analysed the expression of key glycolytic markers (i.e., HKII, LDH‐A and MCT4) by performing immunohistochemistry (IHC) analysis on a tissue microarray (TMA) containing 80 tissue specimens from male and female melanoma patients (with a similar distribution among age and clinical tumour stage). Interestingly, glycolytic markers appeared to be more expressed in male specimens than in female ones (Fig. 1A–C). Coherently, *in silico* analysis of a TCGA melanoma dataset (*n =* 448 patients) revealed that the expression of *LDH‐A*, *HK2* (encoding gene for HKII) and *SLC16A3* (encoding gene for the transporter MCT4) inversely correlate with oestrogen‐related receptor alpha (*ERRα*), a representative female sex marker (Fig. 1D–F). To further corroborate these results, we analysed a clinical database of 114 melanoma patients (45 females and 69 males), and we observed higher LDH serum levels in male patients with respect to female ones (Fig. 1G,H).

To validate the metabolic differences observed in melanoma patient samples in *in vitro* models, we adopted two cell lines derived from male (SkMel28, SSM2c) and female (A375M6, A375P) melanoma patients. In accordance, we found that both male cell lines display higher levels of LDH‐A, HKII and MCT4 (Fig. 1I) and secreted lactate (Fig. 1J) compared to the female counterparts. Also, Seahorse analysis confirmed that male‐derived cells exhibit higher glycolytic basal activity than female ones (Fig. 1K).

### Lactate elicits an enrichment in Treg immunosuppressive infiltration in male melanoma

It is already known that elevated levels of secreted lactate contribute to immunosuppression in the TME, favouring regulatory CD4^+^ T cells (Treg) and M2‐polarised macrophages enrichment [11, 23].

**Fig. 2:**
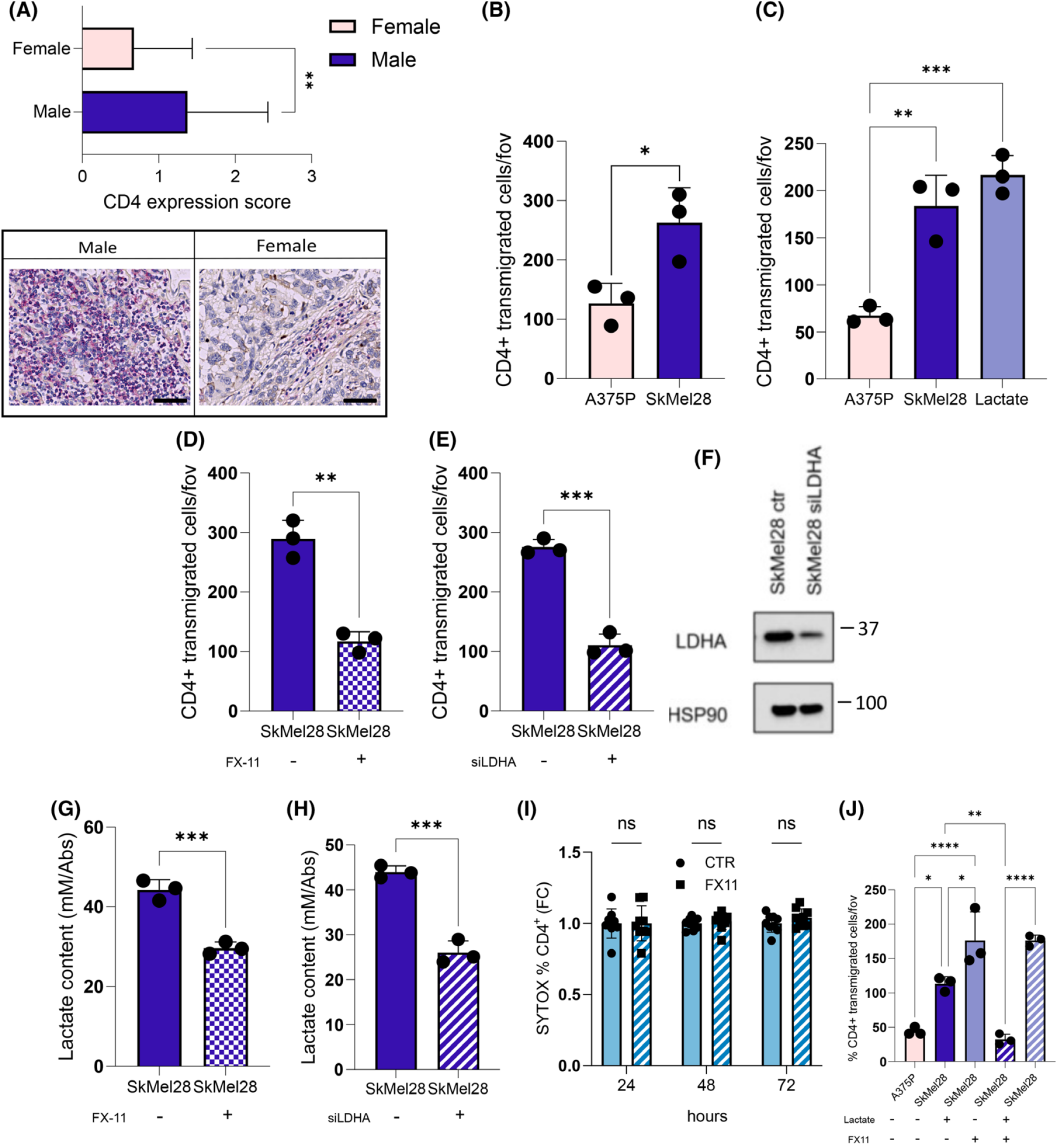
Lactate enhances CD4^+^ T cell recruitment in male‐derived melanoma. (A) Expression score of CD4 in representative tissue cores from TMA (magnification 40×; scale bar: 20 μm) including male and female melanoma samples; *n* = 80. (B) 1.0 × 10^6^ PBMC‐derived CD4^+^ T cells were allowed to migrate towards A375P female and SkMel28 male melanoma cells for 3 h; *n =* 3. (C) 1.0 × 10^6^ PBMC‐derived CD4^+^ T cells were allowed to migrate towards A375P, SkMel28 and lactate (20 mm) for 3 h; *n =* 3. (D, E) 1.0 × 10^6^ PBMC‐derived CD4^+^ T cells were allowed to migrate towards SkMel28 (3 h) in the presence of 10 μm FX‐11 (D) or *LDHA* transiently silenced SkMel28 cells (E); *n =* 3. (F) Representative images of immunoblots for LDH‐A performed in LDHA‐genetic silenced SkMel28 cells using siCTR‐treated cells as comparators. HSP90 was used as loading control. (G, H) Extracellular lactate levels measured by enzymatic assay upon 48 h of co‐culture of CD4^+^ cells with SKMel28 cells treated with 10 μm FX‐11 (G) or transiently silenced for LDH‐A (H); *n* = 3. (I) Viability of CD4^+^ T cells treated with or without 10 μm FX‐11 for 24, 48 and 72 h. Cells were stained with SYTOX Green and analysed by flow cytometry. Data are presented as fold change relative to controls; *n* = 9. (J) 1.0 × 10^6^ PBMC‐derived CD4^+^ T cells were allowed to migrate towards sub‐confluent A375P or SkMel28 (3 h) in presence or absence of lactate, 10 μm FX‐11 and both treatments on SkMel28. Six randomly chosen fields were used for quantification; *n =* 3. Data information: error bars represent means ± SEM of *n* independent experiments. Statistical analysis was performed using two‐tailed unpaired Student's *t*‐test (A, B, D, E, G, H), one‐way ANOVA followed by Tukey's test (C, J) and two‐way ANOVA followed by Sidak's multiple comparisons test (I). ns, not significant; *P < 0.05; **P < 0.005; ***P < 0.001; ****P < 0.0001.

By performing IHC analysis on melanoma patients‐derived TMA, we observed that male samples are characterised by a higher CD4^+^ infiltration than female ones (Fig. 2A). In addition, we found that CD4^+^ T cells, isolated from peripheral blood mononuclear cells (PBMCs) of healthy donors, are more efficiently recruited by SkMel28 male melanoma cells *in vitro*, compared to female A375P cells (Fig. 2B). To investigate whether the metabolic differences observed between male and female melanoma cells could affect the immune infiltration, we explored the role of lactate, more pronounced in male melanoma cell lines, in favouring CD4^+^ T cells recruitment and Treg polarisation. Firstly, lactate is able to efficiently recruit CD4^+^ T cells similarly to that which was observed in SkMel28 male melanoma cells (Fig. 2C). Interestingly, targeting LDH‐A in SkMel28 male melanoma cells by silencing or by administration of the selective inhibitor FX‐11 resulted in a significant reduction in the migration of CD4^+^ cells towards SkMel28 male melanoma cells (Fig. 2D–F), as a result of a decreased tumoural lactate release (Fig. 2G,H), without a toxic effect induced by FX‐11 treatment on CD4^+^ T cells (Fig. 2I). The addition of lactate to FX‐11‐treated SkMel28 male cells restores CD4^+^ T‐cell migration impaired by FX‐11 treatment (Fig. 2J).

**Fig. 3:**
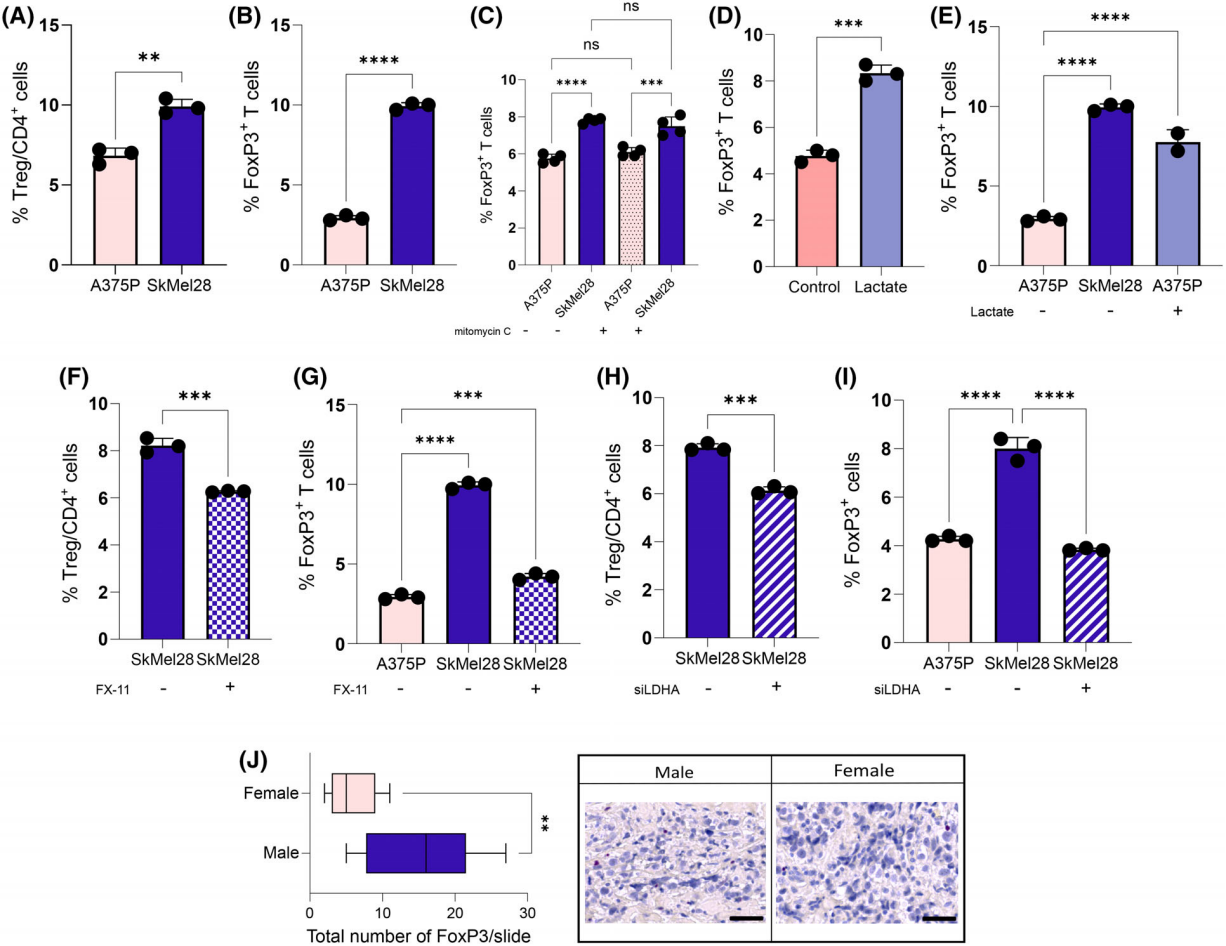
Lactate enhances Treg infiltration in male‐derived melanoma. (A, B) 1.0 × 10^6^ CD4^+^ T cells were co‐cultured for 72 h with female (3 × 10^4^ A375P) or male (3 × 10^4^ SkMel28) melanoma cells. Treg percentage was assessed by flow cytometry as CD4^+^CD25^+^CD127^−^ (A) or CD45^+^CD4^+^FoxP3^+^ cells (B); *n =* 3. (C) 1.0 × 10^6^ CD4^+^ T cells were co‐cultured for 72 h with female (3 × 10^4^ A375P) or male (3 × 10^4^ SkMel28) melanoma cells, in the presence or absence of 0.5 μm mitomycin C. Treg percentage was assessed as FoxP3^+^ cells; *n =* 4. (D) 1.0 × 10^6^ PBMC‐derived CD4^+^ T were cultured in the absence or presence of lactate. Tregs percentage was assessed as CD45^+^CD4^+^FoxP3^+^; *n* = 3. (E) 1.0 × 10^6^ CD4^+^ T cells were co‐cultured for 72 h with female (3 × 10^4^ A375P) melanoma cells, with or without 20 mm lactate or with male (3 × 10^4^ SkMel28) melanoma cells. Tregs percentage was assessed as CD45^+^CD4^+^FoxP3^+^; *n =* 3. (F–I) CD4^+^ T cells were co‐cultured as above (F, G) in the presence (for SkMel28) of 10 μm FX‐11 (F, G), or *LDHA* transiently silenced male SkMel28 cells (H, I). Treg percentage was assessed as CD4^+^CD25^+^CD127^−^ (F–H) or CD45^+^CD4^+^FoxP3^+^ cells (G–I); *n =* 3. (J) Representative IHC detection of FoxP3 in male and female melanoma (magnification 40×; scale bar: 20 μm). Data information: error bars represent means ± SEM of *n* independent experiments. Statistical analysis was performed using two‐tailed unpaired Student's *t*‐test (A, B, D, F, H, J), and one‐way ANOVA followed by Tukey's test (C, E, G, I). ns, not significant; *P < 0.05; **P < 0.005; ***P < 0.001; ****P < 0.0001.

Interestingly, upon CD4^+^ co‐culturing with SkMel28 male cells we observed an enrichment in Treg cells, characterised as both CD4^+^ CD25^+^CD127^−^ and FoxP3^+^ T cells (Fig. 3A,B), suggesting the ability of SkMel28 cells to promote Treg cell polarisation, without affecting cell proliferation (Fig. 3C). Notably, lactate alone increases the percentage of FoxP3^+^ cells *per se* (Fig. 3D), as well as the lactate addition to A375P female cells recapitulating the effects induced by SkMel28 male cells (Fig. 3E), further corroborating the specific role of secreted lactate in inducing Treg cell polarisation. As above, LDH‐A genetic and pharmacological impairment significantly counteracted Treg polarisation by SkMel28 male cells (Fig. 3F–I).

In line with our *in vitro* results, IHC analysis performed on melanoma patient samples revealed higher expression of FoxP3 in male samples with respect to female (Fig. 3J). Taken together, these data underpin the role of LDH‐A and secreted lactate, characterising male melanoma, in fostering CD4^+^ T cell polarisation towards a pro‐tumoural Treg subpopulation.

### Infiltration of functional CD8^+^ T cells is higher in low‐lactate female melanoma

Considering the importance of CD8^+^ T cells in the immunosurveillance in melanoma [22, 24], we also investigated the sex‐ and metabolic‐dependence of CD8 infiltration in melanoma.

**Fig. 4:**
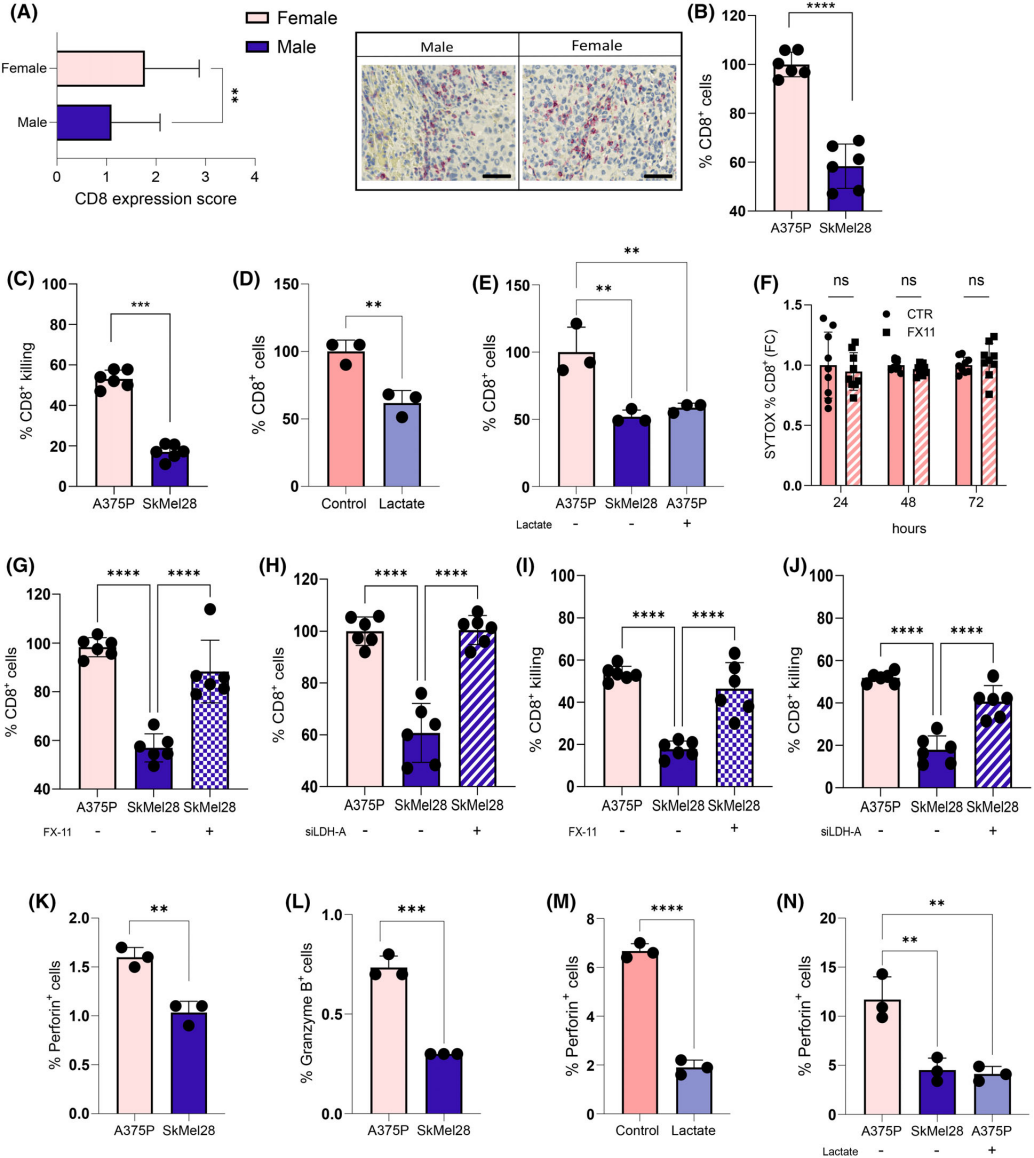
Lower levels of lactate in female melanoma correlate with elevated CD8^+^ T cell abundance. (A) Expression of CD8 in representative tissue cores from TMA (magnification 40×; scale bar: 20 μm) including male and female melanoma samples; *n* = 80. (B) 1.0 × 10^6^ PBMC‐derived CD8^+^ T cells were co‐cultured for 72 h with 3 × 10^4^ melanoma cells (A375P and SkMel28). CD8^+^ T cells were counted and analysed in percentage; *n =* 6. (C) 1.0 × 10^6^ CD8^+^ T cells were co‐cultured for 72 h with 3 × 10^4^ melanoma cells and CD8 killing ability on melanoma was evaluated; *n =* 6. (D) 1.0 × 10^6^ PBMC‐derived CD8^+^ T were cultured in the absence or presence of lactate (20 mm) and the percentage of CD8^+^ T cells was evaluated; *n* = 3. (E) 1.0 × 10^6^ CD8^+^ T cells were co‐cultured for 72 h with female melanoma cells (3 × 10^4^ A375P), with or without lactate (20 mm), or with the male counterpart (3 × 10^4^ SkMel28), and the percentage of CD8^+^ T cells was quantified; *n =* 3. (F) Viability of CD8^+^ T cells treated with or without 10uM FX‐11 for 24, 48 and 72 h. Cells were stained with SYTOX Green and analysed by flow cytometry. Data are presented as fold change relative to controls; *n* = 9. (G, H) 1.0 × 10^6^ PBMC‐derived CD8^+^ T cells were co‐cultured for 72 h with 3 × 10^4^ SkMel28 in presence of 10 μm FX‐11 (F) or with *LDHA* transiently silenced SkMel28 cells (G). CD8^+^ cells were counted and analysed in percentage; *n =* 6. (I, J) 1.0 × 10^6^ CD8^+^ T cells were co‐cultured for 72 h with 3 × 10^4^ SkMel28, in the presence of 10 μm FX‐11 (H), or with LDH‐A transiently silenced SkMel28 cells (I) and CD8 killing ability on melanoma was evaluated; *n =* 6. (K, L) 1.0 × 10^6^ CD8^+^ T cells were co‐cultured for 72 h with female (3 × 10^4^ A375P) or male (3 × 10^4^ SkMel28) melanoma cells. Perforin and Granzyme B positive cells were evaluated by flow cytometry as CD8^+^Perforin^+^GranzymeB^+^ cells; *n =* 3. (M) 1.0 × 10^6^ PBMC‐derived CD8^+^ T were treated as in (D) and the percentage of CD8^+^Perforin^+^ cells was evaluated by flow cytometry; *n =* 3. (N) 1.0 × 10^6^ CD8^+^ T cells were treated as in (E) and the CD8^+^Perforin^+^ cells were quantified; *n =* 3. Data information: error bars represent means ± SEM of *n* independent experiments. Statistical analysis was performed using two‐tailed unpaired Student's *t*‐test (A–D, K–M), one‐way ANOVA followed by Tukey's test (E–J, N) and two‐way ANOVA followed by Sidak's multiple comparisons test (F). ns, not significant; *P < 0.05; **P < 0.005; ***P < 0.001; ****P < 0.0001.

IHC analysis performed on TMA indicated a higher percentage of CD8^+^ T cells in female samples than in male ones (Fig. 4A). In accordance, the percentage and killing function of CD8^+^ T cells was enhanced upon co‐culture with A375P female cells, compared to the SkMel28 male counterpart (Fig. 4B,C), suggesting an impairing role of the SkMel28 lactate‐rich conditioning. Also in accordance, lactate treatment resulted in CD8^+^ T cells reduction, both when administered alone (Fig. 4D) or when added to the A375 female cell lines (Fig. 4E), resembling the effects observed with SkMel28 male cells. In addition, we showed that both LDH‐A pharmacological inhibition, displaying no toxic effects on CD8^+^ T cells (Fig. 4F), and genetic interference in SkMel28 cells restores the percentage and killing activity of CD8^+^ T cells, similar to that observed in female ones (Fig. 4G–J). Accordingly, the levels of T cell‐mediated cytotoxicity markers such as Perforin and Granzyme B were higher in CD8^+^ cells co‐cultured with A375P female melanoma cells than in the male counterpart (Fig. 4K,L) and this effect is phenocopied by lactate administration alone or in addition to the A375 female cells co‐culture (Fig. 4M,N).

**Fig. 5:**
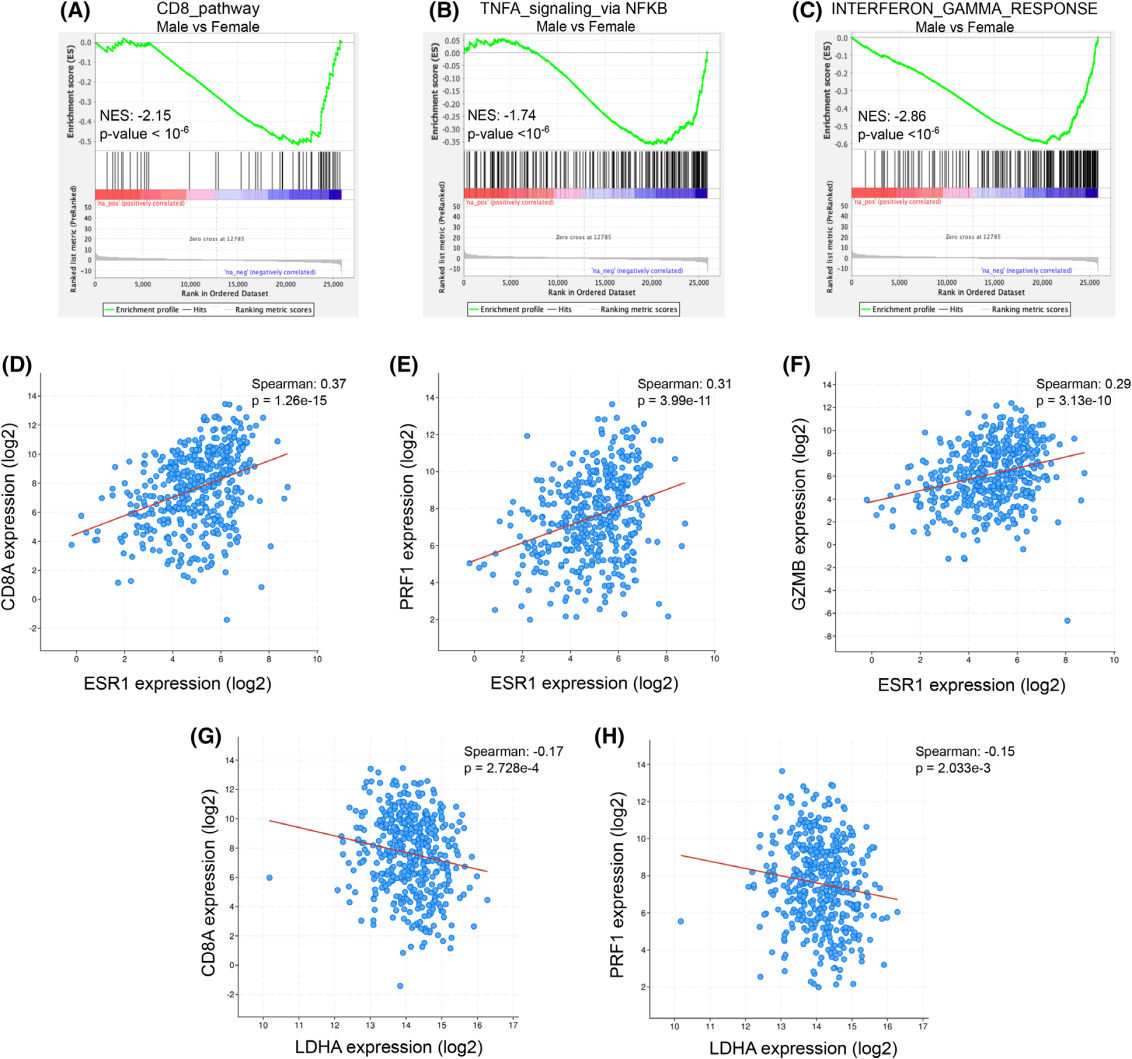
Bioinformatic correlation between metabolic profile and immune infiltration in a sex‐dependent manner. (A) Enrichment plots of the CD8 pathway Hallmark showing a negative NES in male metastatic patients. NES, normalised enrichment score. (B, C) Enrichment plots of the Hallmark TNF‐α and Interferon‐γ negative correlated in male metastatic patients with respect to female ones. NES, normalised enrichment score. (D–F) cBioPortal elucidated positive correlation between *CD8A*, *PRF1* and *GZMB* with *ERRα*; *n* = 448. (G, H) Inverse co‐expression between *CD8A* and *PRF1* with *LDH‐A* as indicated in cBioPortal TCGA melanoma databases; *n* = 448. *P < 0.05; **P < 0.005; ***P < 0.001.

Moreover, GSEA performed on the Hallmark gene sets from the melanoma database highlighted that male patients are negatively associated with gene sets related to the CD8 pathway (Fig. 5A), specifically tumour necrosis factor α (TNF‐α) signaling and Interferon‐γ response, cytokines generally produced by active CD8 cells (Fig. 5B,C), thus reinforcing the sex‐related differences in CD8 percentage and activity.

In agreement, a positive correlation between CD8^+^ T‐cell reactivity markers such as *CD8A* (coding gene for CD8), *PRF1* (as Perforin), *GZMB* (coding gene for Granzyme B) and *ERRα* was observed, as indicated by co‐expression analyses in different melanoma databases (Fig. 5D–F). Moreover, *CD8A* and *PRF1* expression inversely correlate with *LDH‐A*, thereby corroborating that a lactate‐rich environment negatively impacts on the CD8‐mediated antitumour immunity in melanoma (Fig. 5G,H).

These results pinpointed that lower lactate levels in female melanoma favour CD8^+^ immune cell recruitment and activation, finally resulting in a more favourable immune surveillance. Interestingly, interfering with LDH‐A re‐sensitises the CD8‐killing activity in male melanoma cells.

### *In vivo* LDH‐A‐targeting is effective in restoring immune surveillance in melanoma‐bearing male mice, impairing tumour growth and metastatic dissemination

**Fig. 6:**
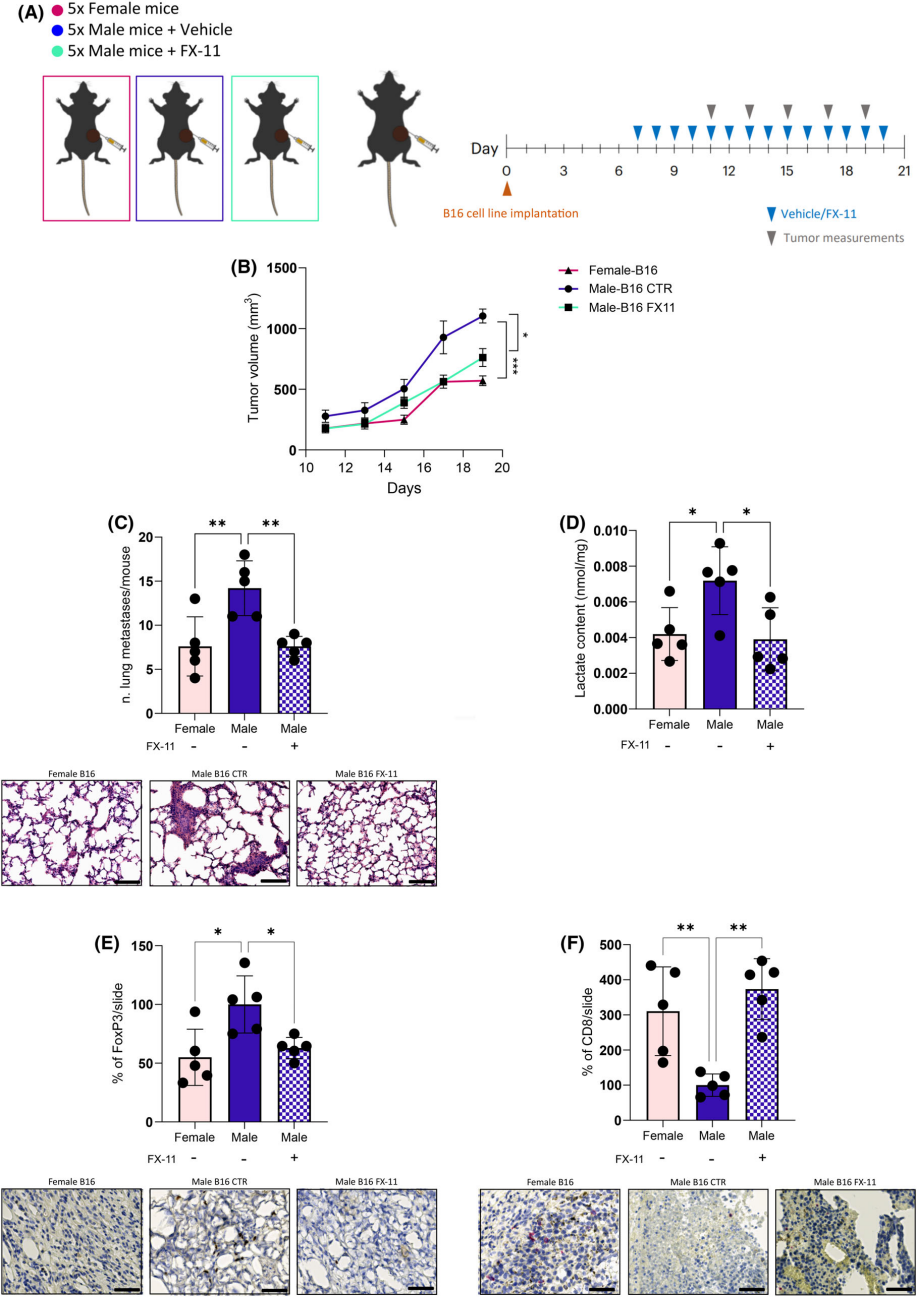
*In vivo* LDH‐A targeting restores immune surveillance and reduces tumour growth and lung colonisation in melanoma‐bearing male mice. (A) 1.0 × 10^6^ B16 cells were injected into the flanks of C57BL/6J female or male mice and, where reported, FX‐11 (2 mg·kg^−1^) or PBS as a vehicle was administered daily. (B) Tumour growth curve for individual mice. Tumour mass was measured at the indicated time after B16 cells implantation and vehicle or FX‐11 treatment; *n = 5*. (C) Quantification of lung metastatic lesions and representative H&E pictures are shown (magnification 20×; scale bar: 40 μm); *n =* 5. (D) Lactate content measured by enzymatic assay in primary tumours derived from the indicated animal cohorts; *n =* 5. (E) Quantification of FoxP3‐staining in primary tumour samples and representative pictures are shown (magnification 40×, scale bar: 20 μm); *n =* 5. (F) Quantification of CD8‐stained detection in primary tumour samples and representative pictures are shown (magnification 40×; scale bar: 20 μm); *n =* 5. Data information: error bars represent means ± SEM of five independent experiments. Statistical analysis was performed using two‐tailed unpaired Student's *t*‐test (B) and one‐way ANOVA followed by Tukey's test (C–F). *P < 0.05; **P < 0.005; ***P < 0.001.

To better characterise the role of LDH‐A in promoting melanoma progression in male individuals and to confirm the efficacy of the pharmacological inhibition of LDH‐A in restoring an immunocompetent tumour environment in a sex‐dependent manner, we took advantage of a syngeneic murine model to establish a melanoma tumour in female and male animals. According to *in vitro* data, we also investigated the effect of LDH‐A‐targeting in male models (Fig. 6A).

Firstly, we observed that a tumour mass generated in male mice was larger than that grown in engrafted females. This was paralleled by an increased number of metastatic lesions in the lung, as a readout of prominent metastatic outgrowth (Fig. 6B,C). Interestingly, LDH‐A‐targeting was highly effective in reducing both primary tumour and metastatic growth in male mice (Fig. 6B,C) and this correlates with a parallel reduction of intra‐tumoural lactate content (Fig. 6D). Together, these data corroborate the strict relationship between sex, glycolytic metabolism, and tumour aggressiveness.

Finally, we investigated the immune infiltration composition in the tumour tissues 20 days post‐injection by IHC analysis. Interestingly, we observed an enrichment in FoxP3^+^ Treg cells with a concomitant reduction in CD8^+^ T cells in male tumours. In agreement with previous results, LDH‐A impairment in male mice dramatically affects both T cell populations (Fig. 6E,F), restoring an immunocompetent microenvironment likely associated with slower tumour growth and decreased metastatic potential.

Overall, these results underscore sex‐related differences in lactate metabolism in melanoma cells, thereby resulting in the establishment of an immunosuppressive *milieu* and an aggressive disease outcome in male individuals. Our data highlight the druggability of this lactate‐driven immune‐metabolic scenario by targeting LDH‐A in a sex‐dependent manner.

## Discussion

Herein, we reported a previously unknown role of lactate as a critical contributor to the sex‐dependent different outcomes of melanoma. The disparities in glycolytic signatures of male and female‐derived melanoma result in a differential enrichment of tumour lactate which orchestrates a Treg‐rich and CD8‐poor immunosuppressive environment in males, contributing to a more aggressive evolution of the disease.

An aberrant metabolism is a distinctive feature of the tumour, which involves the secretion in the extracellular *milieu* of oncometabolites in variable concentrations dependent on different factors, including gender. The metabolic changes occurring in rapidly dividing cancer cells are often closely related to increased glucose intake and abnormal LDH activity, which regulates pyruvate conversion to lactic acid [25]. Moreover, serum LDH levels are commonly increased in cancer patients, including melanoma patients, and correlate with poor clinical outcomes and resistance to therapy [25, 26, 27, 28, 29, 30]. Sex disparities in metabolic pathways can differently contribute to cancer progression, growth and response to anticancer treatments. In this context, it has been previously reported that ERRα activation shows an inverse correlation with LDH‐A in thyroid tumours and skeletal muscle [31], thereby suggesting a sex‐dependent regulation of the tumour metabolic profile.

Recently, Sponagel et al. described sexual dimorphism in metabolite enrichment in brain cancers and demonstrated that male gliomas mainly rely on glutamine uptake. In particular, they demonstrated that GLS1 inhibition reduces the proliferation of tumour male cells by impairing glutamine‐derived glutamate contribution to TCA cycle replenishment [32]. In melanoma, we now have identified sex‐related differences in the glycolytic signature, showing a significant increase in LDH‐A, HKII, and MCT4 levels in male melanoma, further supported by higher serum LDH levels in male patients.

Reducing glycolytic metabolism in tumour cells by either inhibiting glycolytic regulatory enzymes or using competitive glucose analogues (i.e., 2‐DG) has been proposed as an effective strategy to impair tumour progression and potentiate immunosurveillance by repressing tumour infiltrating effector immune cells (i.e., Tregs) expansion and activation [33, 34]. Notably, blocking the recruitment of naïve CD4^+^ T cells allows the reversal of immunosuppression in breast cancer [35].

It is widely reported that Treg cells are one of the primary immune components in the immunosuppressive *milieu*, supporting tumour growth by hindering antitumour immune responses [36, 37, 38]. In a prostate cancer model, lactate released by glycolytic cancer‐associated fibroblasts supports the enhanced tumour malignancy by reducing the presence of Th1 subset cells and increasing the number of pro‐tumourigenic Treg cells [23, 39]. Our data reinforce this evidence, demonstrating that the lactate‐rich environment associated with male melanoma improves CD4^+^ T‐cell recruitment and Treg‐polarisation, and that this is effectively prevented by LDH‐A impairment.

Furthermore, Brand et al. [22] demonstrated that a greater expression of LDH‐A leads to increased lactate production, resulting in a decrease in NK and CD8 cells in melanoma. Accordingly, we observed a higher CD8 killing ability in female samples inversely related to the glycolytic profile, resulting in more effective immune surveillance and a consequent reduction of melanoma progression.

To further validate these sex‐related metabolic and immune differences, we extended our focus to a murine model – highlighting the role of oestrogens in melanoma development – and analysed melanoma patient tissue microarray. These approaches allowed us to confirm and reinforce the metabolic‐immune interactions identified in our cellular models.

In conclusion, our data reported increased LDH‐A expression in male melanoma and suggested a role for lactate in eliciting an immunosuppressive landscape, in a sex‐dependent manner, marked by high Treg‐polarisation and low CD8‐infiltration. Overall, these findings characterise the intimate connection between the glycolytic profile of melanoma and the reshaping of immune infiltration in a specific sex context.

## Materials and methods

### Cell cultures and reagents

Human melanoma cancer cell lines A375P (RRID: CVCL_6233), A375M6 [40] and SkMel28 (RRID: CVCL_0526) were kindly obtained from S. Peppicelli (Department of Experimental and Clinical Biomedical sciences, University of Florence); SSM2c [41] were kindly provided by B. Stecca (ISPRO, Core Research Laboratory, Florence).

Human PBMCs were isolated from adult blood buffy coat samples from healthy donors (obtained from the Azienda Ospedaliera Universitaria Careggi) by Ficoll (Histopaque‐1077; Sigma‐Aldrich, St. Louis, MO, USA) density gradient centrifugation according to manufacturer's procedures. The study methodologies, conforming to the standards set by the Declaration of Helsinki, were approved by the local Ethics Committee (Comitato Etico Regionale per la Sperimentazione Clinica della Regione Toscana) with informed consent from each subject. Briefly, total CD4^+^ T lymphocytes were obtained from PBMCs through a positive selection with antibody‐coated beads according to manufacturer's procedures (#130‐045‐101; Miltenyi Biotec, Bergisch Gladbach, Rhineland, Germany). CD8^+^ T cells were isolated via a positive selection from PBMCs using CD8 MicroBeads according to the kit instructions (#130‐045‐201; Miltenyi Biotec). T cells were maintained in RPMI‐1640 containing 10% FBS (#ECB9006L; Euroclone, Pero, MI, Italy) supplemented with 2 mmol·L^−1^ l‐glutamine and 1% penicillin/streptomycin. All other cells were cultured in phenol‐red Dulbecco's Modified Eagle's Medium (DMEM, #ECB7501L; Euroclone) supplemented with 10% FBS (#ECS5000L; Euroclone), 2 mmol·L^−1^ l‐glutamine (#G7513‐100ML; Merck Sigma, Darmstadt, Germany) and 1% penicillin/streptomycin (#P0781‐100ML; Merck Sigma). All cell lines were cultured at 37°C and 5% CO_2_ and were routinely tested for Mycoplasma contamination with the MycoAlert Mycoplasma Detection kit (#LOLT07710; Lonza – Euroclone).

The FX‐11 inhibitor (##CAS213971‐34‐7; MedChemExpress, Bergkällavägen, Sweden) was dissolved in Dulbecco's phosphate‐buffered saline (PBS) (#ECB4004L; Euroclone) and used at 10 μm in cell culture models. Mitomycin C (#M0503; Sigma‐Aldrich) was dissolved in PBS and used at 0.5 μm in cell co‐cultures.

SYTOX Green nucleic acid stain (#S7020; Thermo Fisher Scientific, Waltham, MA, USA) was used at a final concentration of 20 nm.

Lactic acid (#L1750, Sigma‐Aldrich) was dissolved in water and used at 20 mm.

The following antibodies were used in this study: LDH‐A (1 : 1000 – #GTX10141; GeneTex, Irvine, CA, USA), HKII (1 : 500 – #ab209847; Abcam, Cambridge, UK), MCT4 (1 : 500 – #ab244385; Abcam), HSP90 (1 : 1000 – #sc‐69703; SantaCruz Biotechnology, Dallas, TX, USA), CD4 (1 : 500 – #ab183685; Abcam), FoxP3 (1 : 100 – #ab215206; Abcam), CD8 (1 : 500 – #ab237709), CD8 (1 : 100 – #ab217344; Abcam).

### *In vivo* experiments

Six‐week‐old male and female C57BL/6J mice (Charles River) were hosted at the Centro Stabulazione Animali da Laboratorio (CESAL, Florence, Italy). Mice were kept in standard cages, under specific pathogen‐free conditions with normal dark/light cycle and free access to standard chow (no specific diet) and water. C57BL/6J mice (*n =* 5 for each group) were subcutaneously inoculated with B16 cells (1 × 10^6^ cells), resuspended in 100 μL of PBS (Euroclone) in a double flank injection. Male mice were randomised into two groups and intraperitoneally treated with FX‐11 2 mg·kg^−1^ in 100 μL or vehicle (PBS) once daily. Tumour volume was monitored from day 11 every two days by a digital caliper. Volumes were calculated according to the formula: Volume = (length × width^2^) ÷ 2. The mice have been monitored for the development and growth of primary lesions and sacrificed according to the 3Rs principle. Animals were culled on day 21 when tumour volumes reached the maximum tumour size allowed by Animal Welfare. Primary tumours and metastatic lesions were collected and examined by histological analysis. Tumours and lungs (for the quantification of metastatic colonies) were harvested, formalin‐fixed and paraffin‐embedded for immunohistochemical analysis. Animal work was approved by the Ministero della Salute (License no. 17E9C.265). All the procedures were conducted in accordance with the protocols of the Institutional Animal Care and Use Committee of University of Florence.

### Cell treatment and transfection

SkMel28 male melanoma cells were seeded into 6, 12 or 24‐well plates (75 000, 30 000, 15 000 per well/plates, respectively) to achieve 70% confluence the following day, when cells were transfected with either 20 nm siRNA pool targeting LDH‐A (#EHU116391; Sigma‐Aldrich), or the respective negative controls (#SIC001, siCTR; Sigma‐Aldrich, St. Louis, MO, USA) using Lipofectamine RNAiMAX Reagent (#13778‐150; Thermo Fisher Scientific) and Opti‐MEM (#31985062; Thermo Fisher Scientific) according to manufacturer's instructions. Alternatively, cells were treated with FX‐11 10 μm or mitomycin C 0.5 μm (#M0503; Sigma‐Aldrich). The functional analyses were performed 72 h after transfection as described in the figure legends.

### Cell lysis and Western blot

Melanoma cells were washed with PBS and lysed for 20 min on ice in RIPA lysis Buffer (#89900; Thermo Fischer Scientific) and samples were then boiled at 95°C with 4× Laemmli Sample Buffer (#1610747; Bio‐Rad, Segrate, MI, Italy). Protein concentration was measured by BCA Kit (#23227; Thermo Fisher Scientific). Samples (20 μg) were loaded on 4–20% acrylamide precast SDS/PAGE gels (#456‐8096; Bio‐Rad) and then transferred onto a polyvinylidene difluoride membrane with a Trans‐Blot Turbo Transfer Pack (#1704157; Bio‐Rad). The membranes were activated with methanol and then incubated in 5% non‐fat‐dry milk with the appropriate antibodies overnight at 4°C. The primary antibodies used in this study were reported in the Reagents section with their work concentrations. Membranes were then incubated with the corresponding secondary antibodies for 1 h at room temperature and visualised with Amersham AI600 Chemiluminescent Imager (Amersharm, Heights, IL, USA).

### Database LDH serum

Lactate dehydrogenase serum levels from unresectable stage III and stage IV melanoma patients were kindly provided from Istituto Nazionale bv i IRCCS Fondazione Pascale. A cohort of 114 melanoma patients (45 female and 69 male) was analysed associating normal or high LDH values considering 240–480 U·L^−1^ as a reference range.

### IHC

Individual specimens from mouse tumours/lungs were dissected and fixed in 4% paraformaldehyde for histology and IHC analysis. For human melanoma analysis, we used a tissue microarray (TMA; #MEL1021; Pantomics, Fairfield, CA, USA) that consists of 102 cores comprising 80 cases of melanoma and 22 cases of normal and non‐melanoma tumour tissues of the skin.

Staining of melanoma TMA sections were performed for hematoxylin and eosin (H&E), and the sections were immune‐stained with a panel of antibodies against LDH‐A, HKII, MCT4, CD4, FoxP3 and CD8.

IHC was performed using the Leica BOND‐MAX automated system (Leica Microsystems, Buccinasco, MI, Italy). Murine FoxP3 slides were developed with 3,3‐diaminobenzidine (DAB); LDH‐A, HKII, MCT4, CD4, FoxP3 (human) and CD8 (human/murine) with Fast Red, both counterstained with hematoxylin, dehydrated and mounted. Staining score for primary antibody (LDH‐A, HKII, MCT4, CD4 and CD8 on TMA) was independently analysed by at least two researchers and given a different score, defined as 0 for negative, 1 for weak, 2 for moderate, 3 for strong. The expression of FoxP3 and CD8^+^ T cells was evaluated using the quantification of positive cells in the *in vivo*‐derived tumour sections. Briefly, the number of positive cells were counted at high magnification (40×) and the number of positive cells per field was reported. The antibody specificity was established with a negative and positive human tissue specimen. Metastatic nodules were quantified on each H&E paraffin section with an Aperio Scanner. The sum of microscopic counting was taken as the final number of lung metastatic burden. All images were captured through a slide scanner (Aperio LV1; Leica Biosystems) and analysed using the software imagescope (ImageScope, Leica Biosystems).

### Enzymatic lactate assay

Lactate was quantified from *in vitro* culture medium or melanoma mouse tumour samples. Culture medium of melanoma cells was collected after 48 h culturing and lactate secreted in the medium was quantified by Lactate Assay Kit (#MAK064; Sigma‐Aldrich) in accordance with the manufacturer's instructions. Results were normalised on the total protein concentration of each sample. Intra‐tumoural lactate was evaluated, starting from snap‐frozen tissue samples minced. Melanoma samples were processed with buffer provided by #MAK064 kit using manufacturer's instructions. The nmol obtained from each sample was normalised on tissue mg.

### Seahorse – glycolytic rate assay

A375P, A375M6, SkMel28 and SSM2c cells were seeded in Xfe96 cell culture plates with 1.5 × 10^4^ cells per well in culture medium and incubated overnight at 37°C. Twenty‐four hours post‐seeding, and an hour before assay, standard medium was replaced with bicarbonate‐free (BF) medium (200 μL/well) and cells were allowed to equilibrate in a non‐CO_2_ incubator. Then, basal glycolysis was real‐time measured with Seahorse Xfe96 plate reader (Agilent Technologies, Santa Clara, CA, USA) in basal conditions and upon the supplementation with the respiratory complex I inhibitor rotenone (0.5 μm, for calculation of mitochondrial acidification) and 2‐DG (50 mm, to inhibit glycolysis). Protein quantification was used to normalise the results.

### Database analysis – cBioPortal

We used the cBioPortal online platform to query the TCGA melanoma sequencing dataset (http://www.cbioportal.org). A total of 448 tumour samples (from the TCGA, PanCancerAtlas database – 169 female and 274 male melanoma patients) and 469 patients (from the TCGA, Firehose Legacy) with mRNA next‐generation sequencing data were used. Co‐expressed genes with a significant *P*‐value were studied by cBioPortal online analysis. The following comparisons were analysed: LDHA vs ESR1; HK2 vs ESR1; SLC16A3 vs ESR1; ESR1 vs CD8A‐PRF1‐GZMB; LDHA vs CD8A‐PRF1. Co‐expression data was exported as a graph in *Log scale* and with *Regression line*.

### Differential expression analysis and GSEA

Raw counts of RNA expression, along with patient clinical annotations, were downloaded from TCGA and re‐analysed. RNA‐Seq analysis was carried out using the deseq2 pipeline in an R statistical environment (deseq2 v1.36.0 [42], r v.4.2.1 [43]). Differential expression analysis was performed using the independent filtering procedure to discard genes expressed at low levels, and the computed *P*‐values were adjusted for multiple testing using the Benjamini–Hochberg (FDR) correction method. The subsequent gene‐set enrichment analysis was performed with gsea software [44] in pre‐ranked mode, where input genes were sorted according to the Wald statistic. To identify significantly modulated pathways, the threshold of the *P*‐value was set to 0.05, and the Normalised Enrichment Score (NES) was adopted to figure out direction and magnitude of change.

### Co‐culture of CD4^+^ or CD8^+^ T cells and melanoma cell lines

CD4^+^ T cells (1 × 10^6^) were isolated as described above and co‐cultured with A375P or SkMel28 (3 × 10^4^) in RPM1 1640 medium with or without genetic (si‐LDH‐A) or pharmacological impairment (FX‐11) on cancer cells for 72 h.

CD8^+^ T cells (1 × 10^6^) were isolated as described above and co‐cultured with A375P or SkMel28 (3 × 10^4^) in RPM1 1640 medium with or without genetic (si‐LDH‐A) or pharmacological impairment (FX‐11) on cancer cells for 72 h.

### Dead cell assay

T cell viability was assessed labelling cells with SYTOX Green nucleic acid stain (#S7020; ThermoFisher Scientific), as a dead cell indicator, for 24, 48 and 72 h after FX‐11 treatment. Briefly, 1 × 10^6^ CD4^+^ or CD8^+^ cells were incubated with 20 nm of SYTOX Green for 15 min and analysed by FACS Canto II flow cytometer (BD Bioscience, Milano, Italy). The percentage of SYTOX Green‐positive (dead) cells was normalised to the vehicle‐treated control for each time point.

### CD4^+^ recruitment

Melanoma cell lines were seeded in a culture medium in the lower chamber of 5‐μm‐pore transwell (#3421; Corning, Glendale, AZ, USA) (1–1.5 × 10^4^/well). After 24 h, 1.0 × 10^6^ CD4^+^ T cells, isolated from PBMCs (as previously reported), were seeded into the upper chamber of Boyden transwell (5 μm‐pore‐size). After 3 h, air‐dried membranes were stained with DiffQuick solution (#726443) and the number of recruited CD4^+^ T cells was evaluated by counting cells present at the lower surface of the filters. Six‐randomly chosen fields were used for quantification.

### FACS analysis

For Treg (CD25^+^CD127^−^) analysis, immune cells, upon co‐culturing with melanoma cells for 72 h, were stained with CD3‐APC‐Vio770 (#130‐113‐136), CD4‐PE‐Vio770 (#130‐113‐227), CD25‐Viobright 515 (#130‐113‐287), and CD127‐APC (#130‐113‐413), and Treg were defined as CD3^+^CD4^+^/CD25^+^CD127^−^. For Treg (FoxP3^+^) analysis, immune cells, upon co‐culturing with melanoma cells for 72 h, were stained with Treg phenotyping kit REAfinity (#130‐122‐994). Briefly, we used CD45‐VioBlue (clone: REA747), CD4‐VioGreen (clone: REA623) and FoxP3^+^‐VioR667 (clone: REA944), all from Miltenyi Biotec. For CD8^+^ analysis, immune cells were visualised with CD8‐APC‐Vio770 (#130‐110‐819) surface marker and cell positivity for Perforin‐PE‐Vio770 (#130‐130‐958) and Granzyme‐B‐PE REA226 (#130‐116‐654) was also evaluated.

All markers were from Miltenyi Biotec and were used according to manufacturer's instructions. Flow cytometry was performed by using a FACS Canto II (BD Biosciences) and data analysis was performed with the flowjo software (FlowJo, Ashland, OR, USA).

### Statistical analysis

Statistical analysis was carried out with graphpad prism v9 (GraphPad Software, Boston, MA, USA). Data are presented as means ± SEM from at least three independent experiments. Statistical comparisons: two‐tailed unpaired Student's *t*‐test, one‐way ANOVA followed by Tukey's correction or two‐way ANOVA followed by Sidak's multiple comparisons test. Statistical significance was considered when *P* < 0.05.

## Conflict of interest

The authors declare no conflict of interest.

## Author contributions

MI: Conceptualisation, Formal analysis, Investigation, Data curation, Visualisation, Writing‐original draft, Writing‐review and editing. GC: Conceptualisation, Formal analysis, Investigation, Data curation, Writing‐review and editing. LI: Formal analysis, Writing‐review and editing. GS: Bioinformatics analysis, Resources, Writing‐review and editing. EPa and EPr: Investigation, Writing‐review and editing. MC, GM and PAA: Resources, Writing‐review and editing. PC: Supervision, Funding acquisition, Conceptualisation, Visualisation, Writing‐review and editing. EG: Project administration, Supervision, Funding acquisition, Conceptualisation, Visualisation, Writing‐original draft, Writing‐review and editing.

## Data Availability

The data that support the findings of this study are available from the corresponding author upon reasonable request. The datasets used in this study are openly available at www.cbioportal.org.
